# Effects of Creatine Supplementation after 20 Minutes of Recovery in a Bench Press Exercise Protocol in Moderately Physically Trained Men

**DOI:** 10.3390/nu15030657

**Published:** 2023-01-28

**Authors:** Luis Maicas-Pérez, Juan Hernández-Lougedo, Juan Ramón Heredia-Elvar, Blanca Pedauyé-Rueda, Ana María Cañuelo-Márquez, Manuel Barba-Ruiz, María del Carmen Lozano-Estevan, Pablo García-Fernández, José Luis Maté-Muñoz

**Affiliations:** 1 Department of Physical Activity and Sports Science, Alfonso X El Sabio University, 28691 Madrid, Spain; 2 Department of Physiotherapy, Faculty of Health Sciences, Camilo José Cela University, 28692 Madrid, Spain; 3 Department of Nutrition and Food Science, Complutense University of Madrid, 28040 Madrid, Spain; 4 Department of Radiology, Rehabilitation and Physiotherapy, Complutense University of Madrid, 28040 Madrid, Spain; 5 IdISSC, Instituto de Investigación Sanitaria del Hospital Clínico San Carlos, 28040 Madrid, Spain

**Keywords:** ergogenic aids, athletic performance, resistance exercise, training support, physical fitness

## Abstract

Background: The aims of this study were to analyse the effect of creatine supplementation on the performance improvement in a bench pressing (BP) strength test of muscle failure and to evaluate muscle fatigue and metabolic stress 20 min after the exercise. Methods: Fifty young and healthy individuals were randomly assigned to a creatine group (*n* = 25) or a placebo group (*n* = 25). Three exercise sessions were carried out, with one week of rest between them. In the first week, a progressive load BP test was performed until the individuals reached the one repetition maximum (1RM) in order to for us obtain the load-to-velocity ratio of each participant. In the second week, the participants conducted a three-set BP exercise protocol against 70% 1RM, where they performed the maximum number of repetitions (MNR) until muscle failure occurred, with two minutes of rest between the sets. After one week, and following a supplementation period of 7 days, where half of the participants consumed 0.3 g·kg^−1^·day^−1^ of creatine monohydrate (CR) and the other half consumed 0.3 g·kg^−1^·day^−1^ of placebo (PLA, maltodextrin), the protocol from the second week was repeated. After each set, and up to 20 min after finishing the exercise, the blood lactate concentrations and mean propulsive velocity (MPV) at 1 m·s^−1^ were measured. Results: The CR group performed a significantly higher number of repetitions in Set 1 (CR = 14.8 repetitions, PLA = 13.6 repetitions, *p* = 0.006) and Set 2 (CR = 8 repetitions, PLA = 6.7 repetitions, *p* = 0.006) after supplementation, whereas no significant differences were seen in Set 3 (CR = 5.3 repetitions, PLA = 4.7 repetitions, *p* = 0.176). However, there was a significant increase in blood lactate at minute 10 (*p* = 0.003), minute 15 (*p* = 0.020), and minute 20 (*p* = 0.015) after the exercise in the post-supplementation period. Similarly, a significant increase was observed in the MPV at 1 m·s^−1^ in the CR group with respect to the PLA group at 10, 15, and 20 min after the exercise. Conclusions: Although the creatine supplementation improved the performance in the strength test of muscle failure, the metabolic stress and muscle fatigue values were greater during the 20 min of recovery.

## 1. Introduction

Creatine (methylguanidine acetic acid) is an amino acid that exists at high concentrations in the skeletal muscle (~95%) and at low concentrations in the testicles (~5%) [1,2]. Approximately two thirds of the intramuscular creatine is phosphocreatine (PCr), and the rest of it is free creatine [3]. The total creatine reserve (PCr + free creatine) in the muscle is 120 mmol·kg^−1^ of dry muscle mass on average, reaching an upper limit of creatine storage of 160 mmol·kg^−1^ of dry muscle mass in most individuals [4,5,6]. Since 1–2% of the intramuscular creatine degrades into creatinine (metabolic subproduct) and is excreted in the urine [4,7], the body needs to replace between 1 and 3 g of creatine per day to maintain the normal creatine reserves (without supplementation), depending on the muscle mass. Approximately half of the daily needs of creatine are obtained from the diet [8], mainly from red meat and shellfish [9,10,11]. The remaining amount of creatine is synthesised endogenously in the kidneys and liver in reactions that are mediated by the amino acids arginine, glycine, and methionine [12,13]. Moreover, the intake of commercially manufactured creatine, with creatine monohydrate being the most common type [3], has gained great interest since the first studies were published 30 years ago, which reported that supplementation with exogenous creatine monohydrate increased the intramuscular reserves of PCr [6], and shortly after, these were related to the increase in exercise performance [14,15]. Currently, creatine is one of the most popular nutritional ergogenic aids among professional and amateur athletes [3,16,17], as it is designed to increase exercise-associated strength and power in high-intensity, short (<30 s) exercise sessions [18]. According to the classification system of dietary supplements of the Australian Institute of Sport (AIS), creatine is a class A supplement based on the evidence of its benefits to sport performance [19]. Therefore, creatine has possibly become one of the most studied dietary supplements in the last 25 years, with most studies focusing on analysing its effects on exercise performance [20]. The majority of the studies published in the scientific literature about creatine supplementation report an improvement in the generation of strength (force output), power, anaerobic threshold, and performance during sprints, as well as an increase in the maximum work capacity and the adaptations to training, both in the upper limbs and in the lower limbs during multiple muscle contractions at a maximum level of effort [1,3,15,16,17,20,21,22,23]. Regarding supplementation plans, one of the most effective protocols to increase the muscle reserves of creatine consists of consuming ~0.3 g·kg^−1^·day^−1^ of creatine monohydrate for 5–7 days, followed by a dose of 3–5 g·day^−1^ to maintain the high reserves [3]. Other studies have suggested a creatine supplementation of 20 g·day^−1^ for 5–7 days in combination with exercise [17,21,22,23]. On the same vein, a recent review concluded that consuming 0.3 g·kg^−1^·day^−1^ of creatine monohydrate for 3–5 consecutive days or 20 g·day^−1^ for 5–7 consecutive days produces a rapid increase in the amount of intramuscular creatine, generating immediate ergogenic benefits. The consumption of smaller amounts of creatine monohydrate (3–5 g·day^−1^) also produces an increase in the muscular creatine reserves for a period of 3–4 weeks [3,20], and it improve the muscular performance [20], although the initial effects on performance using these supplementation doses have less support [3]. In addition to the improvements in performance in a single session using a maximum amount of effort, creatine appears to maintain a top performance in multiple exercise sets, thus improving recovery [20]. Sprint improvements have been reported in cycling [14,24,25,26,27], running [28,29,30,31], and swimming [32]. Regarding the strength protocols in which multiple sets are performed until muscle failure occurs with submaximal loads, improvements have also been reported in muscular performance after short-term creatine supplementation [33,34]. Nevertheless, although these earlier studies associate the improvements in performance with a greater capacity to recover between sets, a few studies have measured the recovery period after performing an intense exercise protocol. Recent studies have measured recovery at 15 min after a multiple-set exercise protocol with bench pressing [35] or a high-interval functional training protocol [36], with the aim of determining the recovery time required to perform another exercise protocol in the same session, reaching levels of execution velocity that are similar to those that were obtained in the first protocol, in order to reduce the residual fatigue and increase the performance capacity. In this sense, it would be interesting to know whether creatine supplementation can help people to recover the initial strength levels after performing an exercise protocol. Therefore, the aims of this study were: (1) to analyse the effect of creatine supplementation on the improvement of performances with a bench press strength protocol compared to those of a control group, and (2) to evaluate the muscle fatigue and metabolic stress generated 20 min after performing the exercise protocol, both in the group that consumed creatine and in the control group.

## 2. Materials and Methods

### 2.1. Study Design

**Figure 1:**
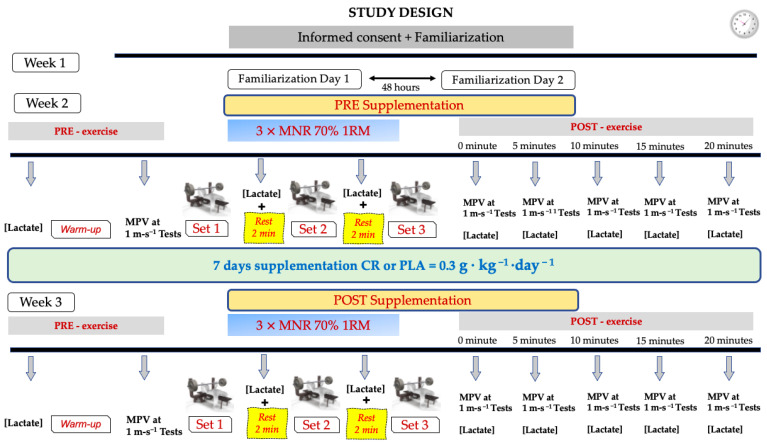
Study design. CR = creatine. PLA = placebo. 1RM = one repetition maximum. MNR = maximum number of repetitions. MPV = Mean Propulsive Velocity.

The present study used a double-blind design with repeated measures, and it was controlled with a placebo group. Three exercise sessions were performed, with one week of rest between them. To obtain the load-to-velocity ratio of each participant, on the first week, a progressive load test was carried out with bench pressing (BP) until the participants reached the one repetition maximum (1RM). In the second week, each participant executed a 3-set exercise with BP, performing the maximum number of repetitions (MNR) until muscle failure occurred, with two minutes of rest between the sets. After a supplementation period of 7 days, during which half of the participants consumed creatine monohydrate (CR), and the other half of them consumed a placebo (PLA), the protocol from the second week was repeated. The measurements were recorded from Monday to Saturday. Each participant performed the first two exercise sessions on the same day of the week, and they carried out the third session on the next day, with an interval of no more than 2 h (±2 h), with the aim of maintaining the same time slot for each participant between pre-supplementation and post-supplementation (cycle of 7 full days starting the supplementation the day after performing the exercise protocol) and controlling the effects of the circadian rhythms [37]. After each set, and up to 20 min after finishing the exercise protocol, the blood lactate concentrations and mean propulsive velocity (MPV) at 1 m·s^−1^ were measured (Figure 1). The entire study was conducted in the exercise physiology laboratory of the university at room temperature (18–23 °C) and a relative humidity of 35–70%.

### 2.2. Participants

**Figure 2:**
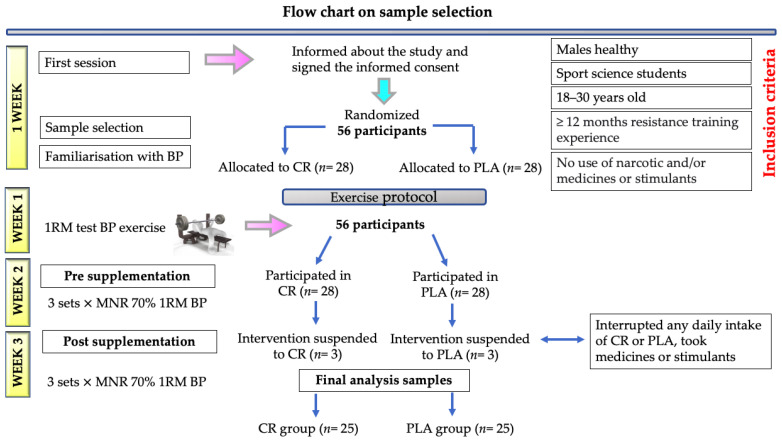
Summary of recruitment, allocation and final analysis samples. CR = creatine. PLA = placebo. BP = bench press. 1RM = one repetition maximum. MNR = maximum number of repetitions.

Fifty-six healthy, male, sports science students were randomly assigned to two experimental groups: half of the participants (*n* = 28) consumed creatine (CR group), and the other half (*n* = 28) of them received a placebo supplement (PLA group). The randomization of participants was achieved in a blinded manner, based on Moses and Oakford’s random permutation table [38]. One week after beginning the study, the participants were gathered and informed about the different aspects related to the study. All of them voluntarily signed the informed consent form. The study included those participants who had at least 12 months of experience in strength training and knew how to execute the BP exercise. Through a questionnaire, the participants reported no musculoskeletal, metabolic, or cardiorespiratory pathologies that could limit their performance. In the same week, and with an interval of 48 h, two sessions were performed to allow the participants to familiarise themselves with the BP exercise. The design of the study, which was approved by the ethics committee of the university, was developed in compliance with the principles of the Declaration of Helsinki [39]. Throughout the 3-week period of the study, the participants were requested not to consume any narcotic and/or psychotropic substances, medicines, or stimulants during the test and supplementation periods. The study excluded those participants who skipped the daily intake of the CR or PLA supplementation at least once. The participants were also requested not to consume any food at least three hours before the tests, allowing them to drink water. All of the individuals were told not to perform any physical exercise or drink alcohol the day before each test. Lastly, since six participants reported that they did not meet some of the inclusion criteria, the sample was reduced to 50 participants (CR, *n* = 25; PLA, *n* = 25) (Figure 2). The GRANMO statistical calculator was used, and we used the standard deviation that had been determined in a previous pilot study with 10 sports science students.

### 2.3. Supplementation

After the first measurement of the protocol of × MNR 70% 1RM, in the second week (pre-supplementation), each participant randomly and double-blindedly received a blank, unlabelled bottle with a supplement of CR (Pure Creatine^®^, Weider Nutrition S.L., Madrid, Spain) or PLA (Pure Maltodextrin^®^, Weider Nutrition S.L., Madrid, Spain). Thus, half of the participants worked under one of the experimental conditions. The CR used in this study was powdered creatine monohydrate, which favours the absorption of creatine [3]. Under normal use conditions and in its original form, the product has no negative effects on health [3]. The placebo used was maltodextrin, which was also powdered, and it was in the same granule size as creatine was. Both of the products were similar in taste, colour (white), texture, and appearance. The dosage was carried out using a dosing device, in which 1 g of product was measured. Neither creatine nor maltodextrin require special storage measures. Two researchers who did not participate in any of the tests were in charge of distributing the bottles and giving the participants the detailed instructions of the supplementation protocol. Based on the guidelines of the International Society of Sports Nutrition, the supplementation dose used for CR was ~0.3 g·kg^−1^·day^−1^ for 7 days [3]. The same dose of maltodextrin was administered to the PLA group. Both the CR dose and the PLA dose were administered in two takes before breakfast and lunch, and they were mixed with water (600 mL). At the end of the study, the participants were asked whether they thought that they had been given creatine or placebo, or whether they were not sure which supplement they consumed. Moreover, the individuals were asked to maintain their habitual dietary intake and physical activity.

### 2.4. Procedures

#### 2.4.1. One Repetition Maximum (1RM) Test

A progressive load test of BP to 1RM (1RM test) was performed based on the warm-up and the execution of the exercise protocol from previous studies [36,40,41], and we obtained the load-to-velocity ratio of each individual.

#### 2.4.2. 3×Maximum Number of Repetitions (MNR) Exercise Protocol

One week after the 1RM test, the exercise protocol was carried out until the participants performed the maximum number of repetitions in each of the three BP sets against 70% 1RM until the muscle failure (MNR) occurred, and we determined this load through the MPV obtained from the individual load-to-velocity ratio in the 1RM test that was conducted in the previous week. The recovery time between sets was 2 min [36,41]. The number of repetitions of each set was recorded, as well as the MPV of the best (fastest) repetition of each set (MPV_rep_ Best), the MPV attained at the last repetition of each set (MPV_rep_ Last), and the loss of the MPV (% loss MPV Set), which is defined as: (MPV_rep_ Last − MPV_rep_ Best)/ MPV_rep_ Best × 100.

#### 2.4.3. Blood Lactate Concentrations

Before the warm-up, 30 s after each of the three sets of exercise and 5, 10, 15, and 20 min after the MNR test, capillary blood samples (5 μL) were extracted from the fingertips of the participants in order to obtain the blood lactate concentrations.

#### 2.4.4. Mechanical Fatigue Test

To measure the mechanical fatigue in the 3 × MNR BP exercise protocol, the percentage of change between the pre-exercise and post-exercise periods was calculated with the load that each individual was able to lift at an MPV of ~1 m·s^−1^ during the propulsive phase (MPV at 1 m·s^−1^ test) based on the test that was conducted in a previous study [42]. This propulsive phase is defined as the concentric phase during which the acceleration of the bar is ≥9.81 m·s^−2^. The value of 1 m·s^−1^ was used due to the fact that it is considered to be a sufficiently high velocity, which can be obtained with BP of moderate intensity or with medium loads (45–50% 1RM). Furthermore, this load is relatively easy to move and well tolerated by any person. The search for the individualised load that was lifted by each of the participants at an MPV of ~1 m·s^−1^ began with a weight of 10 kg, which was increased by 1.25–5 kg, with them performing 3 repetitions with each load and resting for 3 min between the loads. After determining the individualised load at which a velocity of 1 m·s^−1^ was obtained, 3 repetitions of BP were performed before and at 0, 5, 10, 15, and 20 min after the exercise protocol. As per the study by Hernandez-Lougedo et al., [35] the participants performing the same bench pressing protocol almost reached pre-exercise MPV levels after 15 min of recovery, and so a post-exercise measurement at 20 min was included in this study. All of the repetitions were carried out at maximum velocity.

#### 2.4.5. Measurement Equipment

For the 1RM test, the 3 × MNR exercise protocol, and the mechanical fatigue test, a Smith machine with a bar guidance system (Matrix, Chácara Alvorada, Brazil) was used, allowing only the vertical displacement of the bar to occur since the two ends of the bar were fixed. Weight plates of 1.25, 2.5, 5, 10, and 20 kg (Matrix) in weight were employed. The execution velocity of each repetition was estimated using an optoelectronic device, which had previously been validated and calibrated [43]. The sampling frequency of the optoelectronic device was 500 Hz (Velowin v.1.7.232, Instrumentos y Tecnología Deportiva; Murcia, Spain). The software (Velowin v.1.7.232) automatically calculated the MPV using algorithms. The samples of capillary blood lactate were measured using a portable lactate analyser, which had previously been validated and calibrated following the manufacturer’s specifications (Lactate Pro 2 LT-1710, Arkray Factory Inc., KDK Corporation, Siga, Japan) [44,45].

#### 2.4.6. Statistical Analysis

The Shapiro–Wilk test was initially conducted to determine the normality of the variables. Second-order polynomials were employed to establish the load-to-velocity ratio of each individual in the 1RM test. To analyse the different variables in the MNR exercise protocol before and after the supplementation of the two groups (CR and PLA), a two-way ANOVA (group and time) was performed, with repeated measures for the time factor, and we applied Levene’s test to assess the homogeneity of the variances. The effect of the time x group interaction was also analysed by applying Bonferroni’s post hoc index for the pairwise comparison. The statistical power (SP) of the data was also determined, as well as the effect size, which is known as partial eta squared (η_p_^2^), which categorised the magnitude of the differences as trivial (η_p_^2^ ≤ 0.01), small (0.01 ≤ η_p_^2^ < 0.06), moderate (0.06 ≤ η_p_^2^ < 0.14), or large (η_p_^2^ ≥ 0.14) [46]. The sample size was determined by accepting an alpha error of 0.05 and a beta error of 0.15. Fifty-six subjects were required to detect a difference of equal to or greater than 1 unit. A standard deviation of 2.34, which was determined in the previously conducted pilot study, is assumed. A loss to follow-up rate of 12% was estimated. The velocity loss percentage was calculated using the following equation: post − pre/pre × 100. All of the data are expressed as means, standard deviations (SD), and 95% confidence intervals (CI). The significance level was set at *p* < 0.05. All of the statistical tests were performed using the statistical package SPSS version 25.0 (SPSS, Chicago, IL, USA).

## 3. Results

The descriptive statistics and those related to the progressive load test are presented in Table 1.

Table 2 shows the values of the variables related to the MPV of the best repetition, the MPV of the last repetition, and the MPV loss percentage in each set. As they can be observed in this table, significant differences before the supplementation period were only found in the % loss MPV of Set 2 (*p* < 0.05).

**Figure 3:**
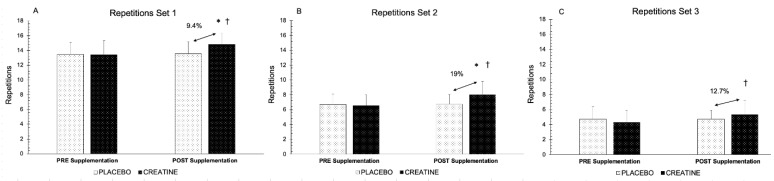
(**A**) Repetitions performed in Set 1 of the protocol of 3 × MNR 70% of MPV in the 1RM test pre- and post-supplementation. (**B**) Repetitions performed in Set 2 of the protocol of 3 × MNR 70% of MPV in the 1RM test in pre- and post-supplementation periods. (**C**) Repetitions performed in Set 3 of the protocol of 3 × MNR 70% of MPV in the 1RM test in pre- and post-supplementation periods. † = significant difference between pre- and post-supplementation periods (*p* < 0.05). MNR = Maximum Number of Repetitions; MPV = Mean Propulsive Velocity. * = significant difference between groups (*p* < 0.05).

Figure 3 represents the number of repetitions performed in each group before and after the supplementation in the BP exercise protocol. Significant differences were obtained in the time x group interaction in Set 1 (*F* (1,48) = 5.566, *p* = 0.022, *η_p_^2^*= 0.104, SP = 0.638) (Figure 3A), Set 2 (*F* (1,48) = 6.440, *p* = 0.014, *η_p_^2^*= 0.118, SP = 0.701) (Figure 3B), and Set 3 (*F* (1,48) = 5.032, *p* = 0.030, *η_p_^2^*= 0.095, SP = 0.594) (Figure 3C). The pairwise comparison through Bonferroni’s post hoc adjustment revealed statistical significance in the CR group between the pre- and post-supplementation periods (Set 1 (pre = 13.4 repetitions, post = 14.8 repetitions), Set 2 (pre = 6.5 repetitions, post = 8 repetitions), *p* = 0.001, and Set 3 (pre = 4.3 repetitions, post = 5.3 repetitions), *p* = 0.003) (Figure 3A–C), while there was no statistical significance between the pre- and post-supplementation periods in the PLA group (Set 1 (pre = 13.5 repetitions, post = 13.6 repetitions) *p* = 0.841, Set 2 (pre = 6.7 repetitions, post = 6.7 repetitions), *p* = 0.921, and Set 3 (pre = 4.7 repetitions, post = 4.7 repetitions), *p* = 1.000). In addition, when we were comparing the CR and PLA groups, there were significant differences between the groups in terms of post-supplementation in Set 1 (CR = 14.8 repetitions, PLA = 13.6 repetitions, *p* = 0.006) and Set 2 (CR = 8 repetitions, PLA = 6.7 repetitions *p* = 0.006) (Figure 3A,B), whereas no significant differences were detected in Set 3 (CR = 5.3 repetitions, PLA = 4.7 repetitions, *p* = 0.176) (Figure 3C). However, there was no statistical significance in the pre-supplementation period in any of the three series (*p* > 0.05).

**Figure 4:**
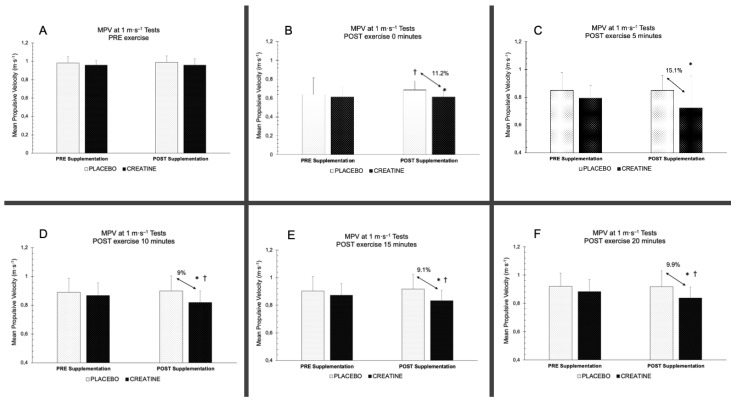
MPV in the 1 m·s^−1^ test in pre- and post-exercise in the CR and PLA groups before and after the supplementation period. † = significant difference between pre- and post-supplementation periods (*p* < 0.05). * = significant difference between groups (*p* < 0.05). MPV = Mean Propulsive Velocity.

Figure 4 shows significant differences between the CR group and the PLA group after the supplementation period in all of the measurements of the MPV in the 1 m·s^−1^ test performed after the exercise (*p* < 0.05). Moreover, for the post-exercise measurements, significant differences were obtained in the time × group interaction at 10 min (*F* (1,48) = 5.681, *p* = 0.021, *η_p_^2^* = 0.106, SP = 0.646)), 15 min (*F* (1,48) = 5.096, *p* = 0.029, *η_p_^2^* = 0.096, SP = 0.600), and 20 min post-exercise (*F* (1,48) = 4.259, *p* = 0.044, *η_p_^2^* = 0.081, SP = 0.525). In the post hoc pairwise comparison, a statistical significance was found in the CR group in the pre- and post-supplementation periods 10 min (*p* = 0.006), 15 min (*p* = 0.025), and 20 min (*p* = 0.017) post-exercise (Figure 4D–F), while in the PLA group, there were no significant differences pre- and post-supplementation periods 10 min (*p* = 0.644), 15 min (*p* = 0.382), and 20 min (*p* = 0.650) post-exercise. Subsequently, when we were comparing the CR and PLA groups, there were significant differences between the groups post-supplementation at 10 min (*p* = 0.004), at 15 min (*p* = 0.002), and 20 min (*p* = 0.002) post-exercise, with no change in the pre-supplementation periods 10 min (*p* = 0.335), 15 min (*p* = 0.272), and 20 min (*p* = 0.160) post-exercise ((10 min, 15 min, and 20 min post-exercise; *p* = 0.335, *p* = 0.272, *p* = 0.160), respectively) (Figure 4D–F).

**Figure 5:**
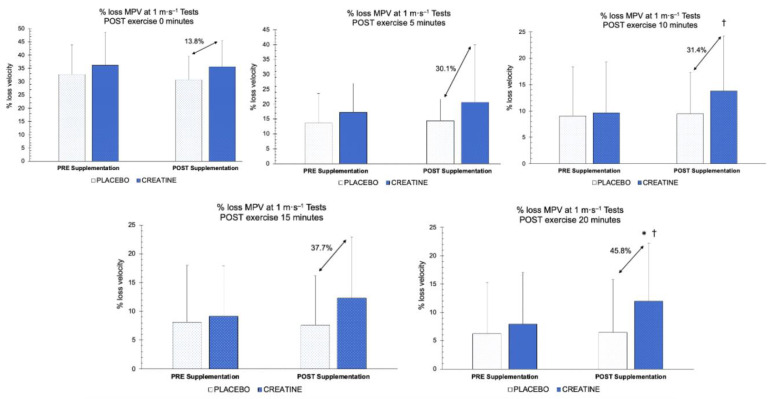
MPV percentage loss in the 1 m·s^−1^ test between groups before and after supplementation. † = significant difference between pre- and post-supplementation periods (*p* < 0.05). * = significant difference between the groups (*p* < 0.05). MPV = Mean Propulsive Velocity.

As it can be observed in Figure 5, the comparison of the MPV loss percentages in the 1 m·s^−1^ test between CR and PLA only revealed significant differences in the time factor at 10 min post-exercise (*F* (1,48) = 4.662, *p* = 0.036, *η_p_^2^*= 0.089, SP = 0.562), whereas no significant differences were obtained in the group factor or in the time × group interaction at any of the post-exercise time points (*p* > 0.05). A post hoc analysis showed significant differences in the CR group at 10 min (*p* = 0.008) and 20 min post-exercise (*p* = 0.028) between the pre- and post-supplementation periods, with no significant differences being found in the PLA group at 10 min (*p* = 0.790) and 20 min post-exercise (*p* = 0.895). Furthermore, while significant differences were also io dentified between the groups at 20 min post-exercise in the post-supplementation period (*p* = 0.05), no such differences were found in the pre-supplementation period (*p* = 0.518).

According to Table 3, no significant differences were detected between the groups in any of the sets of the exercise protocol. However, at 10, 15, and 20 min post-exercise, significant differences were obtained in the group factor and in the time × group interaction (*p* < 0.05). The pairwise comparison revealed greater significant differences in the blood lactate concentrations in the CR group between the pre- and post-supplementation periods at 5 (*p* = 0.020), 10 (*p* < 0.001), 15 (*p* < 0.001), and 20 min post-exercise (*p* = 0.021), while in the PLA group, there were no significant differences pre- and post-supplementation at 5 min (*p* = 0.521), 10 min (*p* = 0.515), 15 min, (*p* = 0.174), and 20 min (*p* = 0.403) post-exercise. Subsequently, when we were comparing the groups (CR and PLA), significantly greater blood lactate concentrations were found in the CR group with respect to the PLA group at 10 (*p* = 0.003), 15 (*p* = 0.020), and 20 min post-exercise (*p* = 0.015) in the post-supplementation period, but no changes were found in the pre-supplementation period 10 min (*p* = 0.503), 15 min (*p* = 0.408), and 20 min (*p* = 0.627) post-exercise.

**Figure 6:**
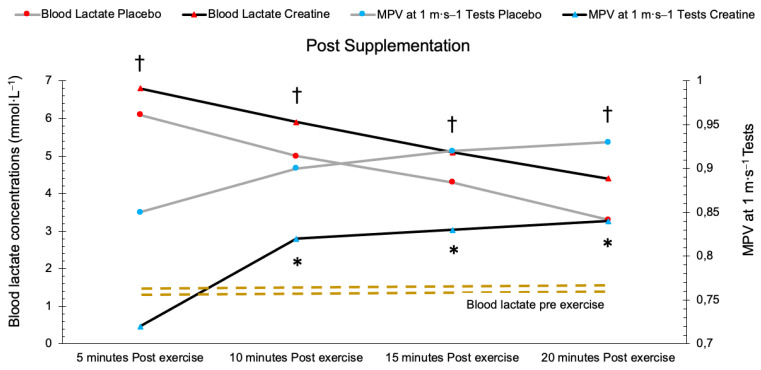
Relation between blood lactate concentration and MPV at 1 m·s^−1^ between the groups in the post-exercise period after the supplementation period. * = significant difference in the MPV at 1 m·s^−1^ test (*p* < 0.05), † = significant difference in blood lactate (*p* < 0.05). MPV = Mean Propulsive Velocity.

Figure 6 shows the blood lactate concentration in relation to the MPV at 1 m·s^−1^ in the post-exercise period between the CR and PLA groups after supplementation. As it can be observed, the group that consumed CR had significantly higher levels of blood lactate in the post-exercise period than those who consumed PLA (*p* < 0.05). Even so, the blood lactate levels in both of the groups are significantly higher than the baseline values (*p* < 0.05). Moreover, the CR group showed a significantly lower MPV values compared to that of the PLA group at 10 and 20 min post-exercise (*p* < 0.05).

## 4. Discussion

The findings of this study indicate that after a dose of 0.3 g·kg^−1^·day^−1^ of creatine monohydrate for 7 days, the muscular performance was increased during repeated BP exercise sets. However, the performance improvement in the group that consumed CR, which resulted in a higher number of repetitions, also increased the metabolic stress and muscle fatigue values with respect to the group that consumed PLA 20 min after the exercise.

This improvement in the MNR performance was observed in all three sets in the CR group, since they performed significantly more repetitions (0.6–1.2 repetitions per set) after the supplementation period, whereas the PLA group maintained the initial values. This explains the significant differences found between the groups in the post-supplementation period in Set 1 and Set 2 of the exercise protocol (9.4% and 19%, respectively). Furthermore, although in Set 3 there was no statistical significance, the CR group performed 12.7% more repetitions. These results are in line with those of previous studies, which consistently report that CR supplementation increases the capacity to recover between peaks of intermittent activity during different types of exercise, such as repeated Wingate efforts [26,47], rowing sprints [48], cycling sprints [14,24,25,49], running sprints [28,29,30,31], swimming sprints [32], resistance exercise [33,34,47], and maximal voluntary isokinetic contractions [15]. This could be due to the increase in the content of intramuscular PCr produced by the CR supplementation, since increases of 10–40% in the content of PCr have been previously reported [6], as well as increases in the amount of PCr re-synthesis during the different recovery periods [50]. These increases would justify the greater content of intramuscular PCr that presented at the beginning of the exercise and, therefore, a greater availability of PCr helps to reduce the decreases in power generation and other fatigue indices during the recovery periods between intermittent anaerobic exercises [20]. In our study, the values of MPV_rep_ Best, MPV_rep_ Last, and the velocity loss percentage in the set are similar before and after the supplementation in both of the groups. However, the CR group maintained the same velocity levels while the participants were performing significantly more repetitions, which represents a performance improvement. Thus, this increase, as has previously been pointed out, would be caused by an increase in the content of intramuscular PCr before each set and after its re-synthesis during the 2 min of recovery between the sets. On the same vein, Yquel et al. [50] stated that the increase in muscle power (~5%) was due to increases in the PCr concentrations, which were accompanied by a lower accumulation of inorganic phosphate and a greater intramuscular pH before people were performing brief periods of maximum exercise (plantarflexion against a resistance corresponding to 66% of the maximum isometric contraction) after 30, 60, and 120 s of recovery. Moreover, the BP exercise protocol that was performed is characterised by large force production, as it is more dependent on type II muscle fibres, which in turn have a greater content of PCr than type I fibres do (which are more oxidative), and thus, they could be favoured by CR supplementation during periods of near-maximum-effort activity [51].

However, the results of the post-exercise recovery for both of the groups show a significant decrease in the MPV at 1 m·s^−1^ for each of the measurements at 5, 10, 15, and 20 min post-exercise in the group that consumed CR, with velocity loss percentages of 11.2%, 15.1%, 9%, 9.1%, and 9.9%, respectively, which represent differences of 0.09 m·s^−1^, 0.13 m·s^−1^, 0.08 m·s^−1^, 0.09 m·s^−1^, and 0.09 m·s^−1^, respectively, compared to those of the placebo group. Moreover, there was a significant decrease in the velocity in the CR group at 10, 15, and 20 min between the pre- and post-supplementation periods. After analysing the velocity loss percentage, statistical significance was only detected between the groups in the post-supplementation period at 20 min post-exercise, with the CR group showing a 45.8% higher velocity loss percentage. This could be related to the fact that the group that consumed CR would present greater muscle fatigue, which decreased the recovery rate with respect to the PLA group. This could be due to the fact that the greater muscle performance during the previous exercise protocol may have generated greater muscle fatigue. In Set 1, the CR group performed 1.2 more repetitions (8.8%); in Set 2, they performed 1.3 more repetitions (19.4%); in Set 3, they performed 0.6 more repetitions (12.8%). On the same vein, a study with well-trained individuals reported a greater power production after a supplementation of 20 g·day^−1^ of CR for 7 days according to 6 × 10 s repeated Wingate tests, which showed a greater fatigue index with respect to the PLA group [26]. However, other studies with supplementation doses of 0.3 g·kg^−1^·day^−1^ of CR for 6 days and 20 g·day^−1^ for 5 days, respectively, did not report changes in the fatigue index in trained cyclists with respect to the PLA group who performed 8 × 15 s bouts of sprint cycling exercise with recovery intervals of 1 and 3 min between the bouts [25] or against a 10 × 6 s Wingate test and a 10 × 40 m sprint test with 24 s of recovery between the sets in well-trained rugby players [52], highlighting that the performance only improved in terms of mean power in the study with the cyclists [25]. Therefore, knowing that the levels of PCr can play an important role as an energy source in the MPV in the 1 m·s^−1^ test, and considering that one of the possible causes of performance improvement could be this increase in the amount of intramuscular PCr, it will be necessary to determine whether there are other metabolic, nervous, or hormonal factors mediating this significantly lower capacity to recover velocity at 10 and 20 min post-exercise.

To the best of our knowledge, this is the first study in the literature that measured post-exercise fatigue through velocity loss in CR supplementation protocols, although recent studies have analysed this mechanical variable. The results of such studies are similar to those of the PLA group, measuring an MPV of 1 m·s^−1^ for up to 15 min after the exercise during the same exercise protocol [35]. Nevertheless, a different study with two types of functional fitness training (strength and endurance ones) involved participants performing squat and military press exercises, and recording an MPV of 1 m·s^−1^ 15 min after the exercises, the researchers found higher velocity values in all of the control points, with them even regaining the pre-exercise velocity after 15 min of recovery [36]. This full recovery of the velocity values could be due to the physical level of the athletes and the difference between the two exercise protocols that were performed (strength and endurance ones). The BP protocol of our study, which was performed until muscle failure occurred, did not enable these participants (with medium strength level: RSR ~1.06) to recover the pre-exercise velocity levels. Thus, it could be inferred that this post-exercise recovery depends on the fitness level of the individual, as well as on the type of exercise that was performed and the muscle groups that were involved.

The analysis of the metabolic stress between the groups shows similar values of blood lactate concentration after all three sets and after 5 min of recovery. This could indicate that subjects who were supplemented with CR, despite them having performed more mechanical work, obtained a similar value of metabolic stress. Previous studies report similar results, with constant blood lactate and pH values, suggesting that anaerobic glycolysis is not altered by CR supplementation [31]. On the same vein, other studies have shown that CR supplementation did not alter the blood lactate concentrations after 3.5 min of recovery following intermittent sprints in soccer players [29], nor immediately or one hour after performing 6 × 35 m sprints at a maximum speed with 10 s of recovery between the sprints [30]. However, in other studies, the blood lactate levels decreased 3 min after bouts of high-intensity cycling after CR supplementation [14,53]. Therefore, it could be assumed that an increase in the rate of muscle PCr re-synthesis in the CR group could have contributed to a better performance in each of the three sets. However, it is important to note that the assessment of metabolic stress is based solely on the blood lactate concentrations and not on other metabolites. Therefore, these results will need to be confirmed by performing further research.

Furthermore, the results obtained from 10 min post-exercise show greater blood lactate concentrations in the CR group, but these differences are not clinically significant. This could be due to the higher number of repetitions performed in each of the three sets, since although the blood lactate levels were similar after each exercise set, they could have increased a few minutes later. Previous studies have also reported significant changes in the blood lactate levels between groups of trained and non-trained individuals 15 and 30 min after the exercise, with here being no changes immediately after conducting the exercise [54]. Although this decrease in the amount of blood lactate corresponded to the trained individuals, since they had a greater lactate storage capacity [54], in our study, the longer time taken to perform more repetitions (in the CR group) may have caused the blood lactate levels to increase significantly a few minutes later (10–15 min post-exercise). This is a new fact that has not been described so far in the scientific literature, and further studies will be necessary to confirm these results. Moreover, the time required to execute each of the sets in the exercise protocol could justify the fact that the main source of energy was glycolysis, since previous studies have reported on the high availability of PCr up to the first 10 s during the exertion of maximum effort, after which most of the ATP produced is due to glycolysis [55]. In addition, it has been shown that 25–30% ATP that has been re-synthesised from anaerobic metabolism comes from PCr after performing a single 30 s cycle ergometer sprint, whereas most of it (65–70%) comes from glycolysis [56,57]. Another study highlights a similar contribution between PCr degradation and anaerobic glycolysis in the exertion of maximum efforts during the first 6 s of very intense exercise [58]. Therefore, although the total time spent performing each set of the exercise was not measured, adding the repetitions that were performed and estimating, on average, that the time between the eccentric phase (~1 s) and the concentric phase (~1 s) may be close to 2 s, the time taken exerting the effort would be over 10 s in all of the sets. Furthermore, if the CR group performed more repetitions, this could justify the fact that the blood lactate concentrations remained constant in the final minutes of recovery with respect to the PLA group.

This study presents some limitations, such as the lack of muscle biopsies and measurements of other metabolites (e.g., muscle PCr, inorganic phosphorus, ammonium, and intramuscular or blood pH), which would help us to understand the mechanisms underlying the improvement of the performance of intermittent efforts until a muscle failure occurred. These parameters would also contribute to a better understanding of why the metabolic and velocity recovery period in the minutes after the exercise is slower after CR supplementation, and they could be used to extrapolate the results of other populations, which would require further research. In addition, future studies are necessary due to the great heterogeneity among the exercise protocols, which could involve different exercise times, types of exercises that need to be performed, muscle groups involved in the exercise, and fitness levels of the participants (elite athletes, trained individuals, and non-trained individuals). In addition, a recent study has described changes in the gut microbiota and immunological factors associated with maltodextrin ingestion as a placebo control substance in human treatment/intervention research [59]. Although this study does not address the effects on sports performance, future research should consider that the use of maltodextrin as a placebo could affect the physical performance of the participants. Although the participants were asked to follow a normal dietary intake, maintaining their eating routines, in no case was food eaten during the study monitored, which is also a limitation of the study.

## 5. Conclusions

To sum up, the group that consumed 0.3 g·kg^−1^·day^−1^ of creatine monohydrate for 7 days improved their performance by carrying out a higher number of repetitions than the placebo group did during the execution of the bench pressing sets until muscle failure occurred. Since anaerobic glycolysis is not altered by supplementation during the exercise protocol (it shows similar values to that of the blood lactate concentration), it is possible that the higher PCr levels in the CR group increases the capacity to recover between the sets of intermittent bench pressing activity. However, during the 20 min in the recovery period, the blood lactate levels were higher, and the velocity values were lower than those of the placebo group.

## Figures and Tables

**Table 1:** Data related to the progressive load test to 1RM.

Variable	(M ± SD) (n = 50)	95%CI
Age (years)	22.40 ± 3.31	21.46–23.34
Weight (kg)	76.89 ± 9.78	74.11–79.67
Height (m)	1.79 ± 0.06	1.77–1.80
BMI (kg·m^−2^)	24.02 ± 2.33	23.36–24.68
RSR (kg at 1RM·kg body mass^−1^)	1.06 ± 0.20	1.00–1.11
1RM (kg)	80.76 ± 16.59	76.05–85.47
MPV 1RM (m·s^−1^)	0.17 ± 0.07	0.15–0.19

BMI = Body mass index; RSR = Relative Strength Ratio, defined as 1RM value divided by body mass; 1RM = one repetition maximum; MPV = Mean Propulsive Velocity. M = mean ± SD = standard deviation; CI = confidence interval.

**Table 2:** Data related to the repetitions of the bench press exercise protocol with a load of 70% MVP obtained in the 1RM test of each individual.

		PRE Suppl	POST Suppl	PRE Suppl	POST Suppl	PRE Suppl	POST Suppl
	Group	MPV_rep_ Best (m·s^−1^) (M ± SD, 95% CI)	MPV_rep_ Best (m·s^−1^) (M ± SD, 95% CI)	MPV_rep_ Last (m·s^−1^) (M ± SD, 95% CI)	MPV_rep_ Last (m·s^−1^) (M ± SD, 95% CI)	% loss MPV Set (M ± SD, 95% CI)	% loss MPV Set (M ± SD, 95% CI)
	CREATINE	0.62 ± 0.09	0.63 ± 0.07	0.14 ± 0.04	0.15 ± 0.04	−77.94 ± 5.82	−75.81 ± 6.49
SET 1		0.58–0.66	0.60–0.66	0.12–0.16	0.13–0.17	−80.87–−75.01	−78.61–−73.01
	PLACEBO	0.60 ± 0.11	0.61 ± 0.08	0.15 ± 0.05	0.16 ± 0.05	–74.11 ± 8.50	–73.88 ± 7.41
		0.56–0.64	0.58–0.64	0.14–0.17	0.14–0.17	−77.04–−71.19	−76.68–−71.08
	CREATINE	0.42 ± 0.07	0.40 ± 0.06	0.17 ± 0.04	0.16 ± 0.04	−59.25 ± 10.36 *	−59.16 ± 9.15
SET 2		0.39–0.45	0.38–0.42	0.15–0.19	0.14–0.18	−63.37–−55.12	–63.03–−55.29
	PLACEBO	0.44 ± 0.07	0.43 ± 0.06	0.14 ± 0.05	0.16 ± 0.05	−67.20 ± 10.15	−63.16 ± 10.07
		0.41–0.47	0.40–0.45	0.13–0.16	0.14–0.18	−71.32–−63.07	−67.03–−59.29
	CREATINE	0.35 ± 0.05	0.34 ± 0.05	0.17 ± 0.06	0.16 ± 0.06	−52.32 ± 15.77	−53.40 ± 20.99
SET 3		0.33–0.37	0.32–0.36	0.15–0.19	0.13–0.18	−58.69–−45.96	−60.27–−46.5
	PLACEBO	0.37 ± 0.06	0.36 ± 0.05	0.16 ± 0.5	0.15 ± 0.04	−53.89 ± 15.88	−58.52 ± 12.01
		0.34–0.39	0.34–0.38	0.14–0.18	0.13–0.17	−69.75–−78.17	−65.40–−51.65

PRE Suppl = Pre-supplementation; POST Suppl = Post-supplementation; MPV = Mean Propulsive Velocity; MPV_rep_ Best = Mean propulsive velocity attained in the best repetition; MPV_rep_ Last = Mean propulsive velocity attained in the last repetition; M = mean ± SD = standard deviation; CI = confidence interval. * = significant difference between groups (*p* < 0.05).

**Table 3:** Data related to the repetitions of the bench press exercise protocol with a load of 70% MVP obtained in the 1RM test of each individual.

		PRE Suppl	POST Suppl			
	Group	Lactate (mmol·L^−1^) (M ± SD, 95% CI)	Lactate (mmol·L^−1^) (M ± SD, 95% CI	*p* Time η_p_^2^ SP	*p* Group η_p_^2^ SP	*p* Group × Time η_p_^2^ SP
Pre-exercise	CREATINE	1.3 ± 0.3	1.4 ± 0.3	0.054 0.075 0.492	0.780 0.002 0.059	0.513 0.009 0.099
		1.2–1.5	1.3–1.6			
	PLACEBO	1.3 ± 0.3	1.5 ± 0.5			
		1.2–1.5	1.3–1.6			
SET 1	CREATINE	4.9 ± 1.2	4.8 ± 0.9	0.993 0.000 0.050	0.675 0.004 0.070	0.838 0.001 0.055
		4.3–5.4	4.4–5.3			
	PLACEBO	4.7 ± 1.5	4.8 ± 1.2			
		4.2–5.3	4.4–5.2			
SET 2	CREATINE	5.7 ± 1.0	6.0 ± 0.7	0.320 0.021 0.167	0.760 0.002 0.060	0.602 0.006 0.081
		5.1–6.2	5.6–6.3			
	PLACEBO	5.9 ± 1.6	5.9 ± 1.0			
		5.3–6.4	5.6–6.3			
SET 3	CREATINE	6.8 ± 1.5	7.0 ± 1.4	0.081 0.062 0.416	0.691 0.003 0.068	0.486 0.010 0.106
		6.2–7.4	6.4–7.5			
	PLACEBO	6.5 ± 1.5	7.0 ± 1.4			
		5.9–7.1	6.4–7.5			
	CREATINE	6.2 ± 1.0	6.8 ± 1.8	0.035 * 0.091 0.565	0.202 0.034 0.245	0.213 0.033 0.236
5 min Post-exercise		5.7–6.7	6.2–7.4			
	PLACEBO	6.0 ± 1.3	6.1 ± 1.2			
		5.5–6.5	5.5–6.7			
10 min Post-exercise	CREATINE	5.1 ± 1.1	5.9 ± 1.1	0.002 * 0.175 0.879	0.043 * 0.082 0.529	0.028 * 0.097 0.604
		4.6–5.5	5.5–6.3			
	PLACEBO	4.8 ± 1.3	5.0 ± 1.0			
		4.3–5.3	4.6–5.4			
15 min Post-exercise	CREATINE	4.1 ± 0.6	5.1 ± 1.7	<0.001 * 0.258 0.980	0.043 * 0.083 0.532	0.038 * 0.087 0.552
		3.8–4.4	4.6–5.7			
	PLACEBO	3.9 ± 1.0	4.3 ± 0.9			
		3.6–4.2	3.7–4.8			
20 min Post-exercise	CREATINE	3.7 ± 1.0	4.4 ± 2.0	0.281 0.024 0.187	0.040 * 0.085 0.545	0.027 * 0.098 0.609
		3.3–4.1	3.8–5.0			
	PLACEBO	3.6 ± 0.8	3.3 ± 0.8			
		3.2–3.9	2.7–3.9			

PRE Suppl = Pre-supplementation; POST Suppl = Post-supplementation; M = mean ± SD = standard deviation; CI = confidence intervals; SP = statistical power; η_p_^2^ = partial eta squared. * = significant difference (*p* < 0.05).

## Data Availability

Not applicable.
